# Sodium butyrate attenuated diet-induced obesity, insulin resistance and inflammation partly by promoting fat thermogenesis *via* intro-adipose sympathetic innervation

**DOI:** 10.3389/fphar.2022.938760

**Published:** 2022-10-03

**Authors:** Wanlong Zhu, Ke Peng, Yan Zhao, Changjing Xu, Xuemei Tao, Yuanzhi Liu, Yilan Huang, Xuping Yang

**Affiliations:** ^1^ Department of Pharmacy, The Affiliated Hospital of Southwest Medical University, Luzhou, China; ^2^ School of Pharmacy, Southwest Medical University, Luzhou, China; ^3^ Department of Nuclear Medicine, The Affiliated Hospital of Southwest Medical University, Luzhou, China

**Keywords:** sodium butyrate, obesity, adipose tissue, thermogenesis, sympathetic innervation

## Abstract

Emerging evidence suggests that butyrate, a short-chain fatty acid, may have beneficial effects on obesity and its associated metabolic comorbidities, but the related molecular mechanism is largely unknown. This study aims to investigate the role of butyrate in diet-induced obesity and metabolic disorders and the relevant regulatory mechanisms. Here, dietary supplementation with Sodium butyrate (NaB) was carried out in mice fed with a high-fat diet (HFD) or chow diet. At week 14, mice on HFD displayed an obese phenotype and down-regulated expression of thermogenic regulators including *Ucp-1* and *Pgc-1α* in adipose tissue. Excitingly, NaB add-on treatment abolished these detrimental effects. Moreover, the obesity-induced insulin resistance, inflammation, fatty liver, and intestinal dysfunction were also attenuated by NaB administration. Mechanistically, NaB can promote fat thermogenesis *via* the increased local sympathetic innervation of adipose tissue, and blocking the β3-adrenergic signaling pathway by 6-hydroxydopamine abolished NaB-induced thermogenesis. Our study reveals a potential pharmacological target for NaB to combat obesity and metabolic disorders.

## Introduction

In recent years, along with the change in dietary habits and sedentary lifestyles, the prevalence of obesity significantly increased (Soltani et al., 2021). Obesity is associated with many chronic diseases such as diabetes, fatty liver, and cancer (Park et al., 2020), which arises from an imbalance between food intake and energy expenditure that eventually leads to an excessive accumulation of fat mass (Kim et al., 2020). Mammalian adipose tissue is generally classified into white and brown adipose tissues. White adipose tissue (WAT) stores energy in the form of triglycerides, and excessive WAT results in obesity and insulin resistance (An et al., 2021). Meanwhile, brown adipose tissue (BAT) consumes energy depending on the mitochondrial uncoupling protein 1 (UCP-1), which dissipates the mitochondrial proton gradient leading to heat generation (Shamsi et al., 2020). Recently, a new type of thermogenic adipocyte, known as beige adipocyte, has emerged in WAT upon cold exposure or β3-adrenergic receptor (β3AR) agonists (Liu et al., 2019; Becher et al., 2021). This process is the so-called “WAT browning” (Becher et al., 2021). Multiple studies have shown that increasing brown and beige fat activity can effectively increase whole-body energy expenditure, which can be used therapeutically to reduce diet-induced obesity and the associated complications (Balazova et al., 2021; Cui et al., 2021).

Adipose tissue is innervated by the sympathetic nervous system (SNS), which plays a key role in controlling its lipolytic and thermogenic functions (Bartness et al., 2014). When exposed to lower temperatures, cold stimulates sympathetic outflow to adipose depots, which acts through secreting neurotransmitter norepinephrine (NE) to adipocytes (Seki et al., 2016). Mechanically, NE binds to β3AR, then activates cyclic adenosine monophosphate (cAMP)-dependent protein kinase A (PKA) (Harms and Seale, 2013), which ultimately promotes the activation of transcriptional factors such as cAMP-response element binding protein (*Creb*), thermogenic markers such as peroxisome proliferator-activated receptor γ coactivator-1α (*Pgc-1α*) and uncoupling protein 1 (*Ucp-1*) (Altarejos and Montminy, 2011). Surgical or pharmacologic destruction of these sympathetic fibers inhibits the leptin-stimulated lipolytic response and cold-induced beiging process of WAT(Zeng et al., 2015; Jiang et al., 2017). Meanwhile, optogenetic activation of sympathetic fibers in fat has been reported to induce lipolysis and reduce fat mass (Zeng et al., 2015). Thus, targeting the sympathetic neurons in fat has the potential to be a novel anti-obesity strategy.

The short-chain fatty acids (SCFAs) including acetate, propionate, and butyrate are products of dietary fiber metabolization by gut microbiota (Luu et al., 2019). SCFAs are widely thought to serve multiple roles in host homeostasis and mediate the beneficial effects of the microbiota community (Zhang et al., 2020). Among SCFAs, butyrate has been studied extensively in immunomodulatory and gut barrier function (Luu et al., 2019). Comparisons of microbiota composition have shown lower abundance of a butyrate-producing bacterium in diabetic or obese patients, while dietary supplementation with butyrate ameliorates inflammation and insulin resistance (Li et al., 2020; Gart et al., 2021). Thus, increasing attention has been paid to butyrate for its protective role in obesity (Coppola et al., 2021). In this study, *Sodium butyrate* (NaB) was found to protect against diet-induced-obesity (DIO) by activating brown fat and white fat browning *via* the increased sympathetic innervation of adipose tissue, thus attenuated the consequent insulin resistance and inflammation which is expected to provide an opportunity for combating obesity and metabolic diseases.

## Materials and methods

### Animals and experimental design

C57BL/6J male mice, 6 weeks old, 20–22 g (HFK Co. Ltd., Beijing, China) were used in this study. All mice were housed in temperature-controlled (23 ± 2°C), pathogen-free barrier facility on a 12 h/12 h light/dark cycle and had access to food and water *ad libitum*. After 1 week of acclimatization, all mice were randomly divided into three groups, six mice per group: control group that received normal chow diet (CD, 1022); obese group that received high-fat diet (HFD, D12492, 60 kcal% fat); NaB-treated group that received HFD with 0.4% w/w of NaB (HNaB). The whole experiment lasted for 14 weeks, then mice were fasted overnight and euthanized under terminal anesthesia. Blood samples, adipose tissue, liver and gut were collected for further experiment. All animal studies were conducted in compliance with the National Institutes of Health guidelines and were approved by the Animal Care and Use Committee of Southwest Medical University.

### Serum biochemistry

Mouse blood samples were allowed to clot at room temperature followed by extraction of serum *via* centrifugation. Serum triglycerides, total cholesterol and glucose were measured using standard enzymatic methods. For serum tumor necrosis factor-α(TNF-α), Lipopolysaccharide (LPS) and insulin measurement, the ELISA kits were used according to the manufacturer’s instructions.

### Hematoxylin-eosin staining

Mouse colon, liver and adipose tissues were fixed with 10% neutral buffered formalin for 24 h, embedded in paraffin blocks. All slices were dewaxed with xylene for 10 min, anhydrous ethanol for 10 min, 90% ethanol for 5 min, 80% ethanol for 5 min and 70% ethanol for 5 min. Tissue histology was evaluated using hematoxylin-eosin staining. Image acquisition was carried out by using a microscope (Olympus America Inc. Melville, NY, United States). Adipocyte cross-sectional area and distribution were determined using ImageJ software (NIH image software).

### Immumohistochemical staining

The waxing and dewaxing of the tissue samples were performed as described previously. Deparaffinized sections were rehydrated and boiled in 10 mM citric acid at 98°C for 10 min, then treated for 20 min with 0.3% H_2_O_2_ in PBS for antigen retrieval. Tissue sections were incubated with 10% normal goat serum for 30 min to block non-specific antibody binding. After washing, primary antibodies were incubated overnight at 4°C (anti-UCP-1, 1:500). The following day, the sections were incubated with the appropriate secondary antibodies. The 3,3-diaminobenzidine (DAB) solution was used as a chromogen. Images were acquired with a microscope connected to a camera (Olympus America Inc. Melville, NY, United States).

### Real-time quantitative PCR

The liver, gut and adipose tissues were homogenized using TissueLyzer and total RNA was isolated using TRIzol Reagent (Vazyme, Nanjing, China). 2–5 ng of cDNA was amplified using Bio-Rad CFX ConnectTM Real-Time System (CFX ConnectTM Real-time system, Bio-Rad, United States) with universal SYBR green mix (Vazyme, Nanjing, China). The thermal profile employed one cycle of initial denaturation at 95°C for 5 min, 39 cycles of denaturation at 95°C for 20 s, annealing at 60°C for 20 s and extension at 72°C for 20 s. Relative expression levels of target genes were converted using the 2^−ΔΔCT^ method against the internal control 18 s, and data were presented as fold change relative to control groups. For primer sequences, refer to Supplementary Table S1.

### Western-blot analysis

Adipose tissues were homogenized with 1×loading lysis buffer. Protein concentrations were detected by a Bradford protein assay kit (Thermo Scientific, United States). Equal amounts of protein were loaded on 10% SDS-PAGE gel and transferred onto a polyvinylidene difluoride (PVDF) membrane (Millipore, Billerica, MA). The membranes were blocked by 5% skim milk for 60 min, and then incubated overnight at 4°C with primary antibody. Membranes were washed and incubated in secondary antibody for 1 h. The ECL chromogenic substrate was used to visualize the bands (Epizyme Biotech, China). Protein bands were quantified using ImageJ software. For primary antibodies, refer to Supplementary TableS2.

### Data and statistical analysis

All statistical analyses were performed using GraphPad Prism eight software (San Diego, CA, United States). All data are presented as mean ± SE. The data were analyzed using one-way analysis of variance (ANOVA) followed by post hoc Tukey’s tests and paired sample *t* test for comparisons between multiple groups. *p* < 0.05 was considered as statistically significant.

## Results

### Effects of NaB on body weight under HFD

**FIGURE 1:**
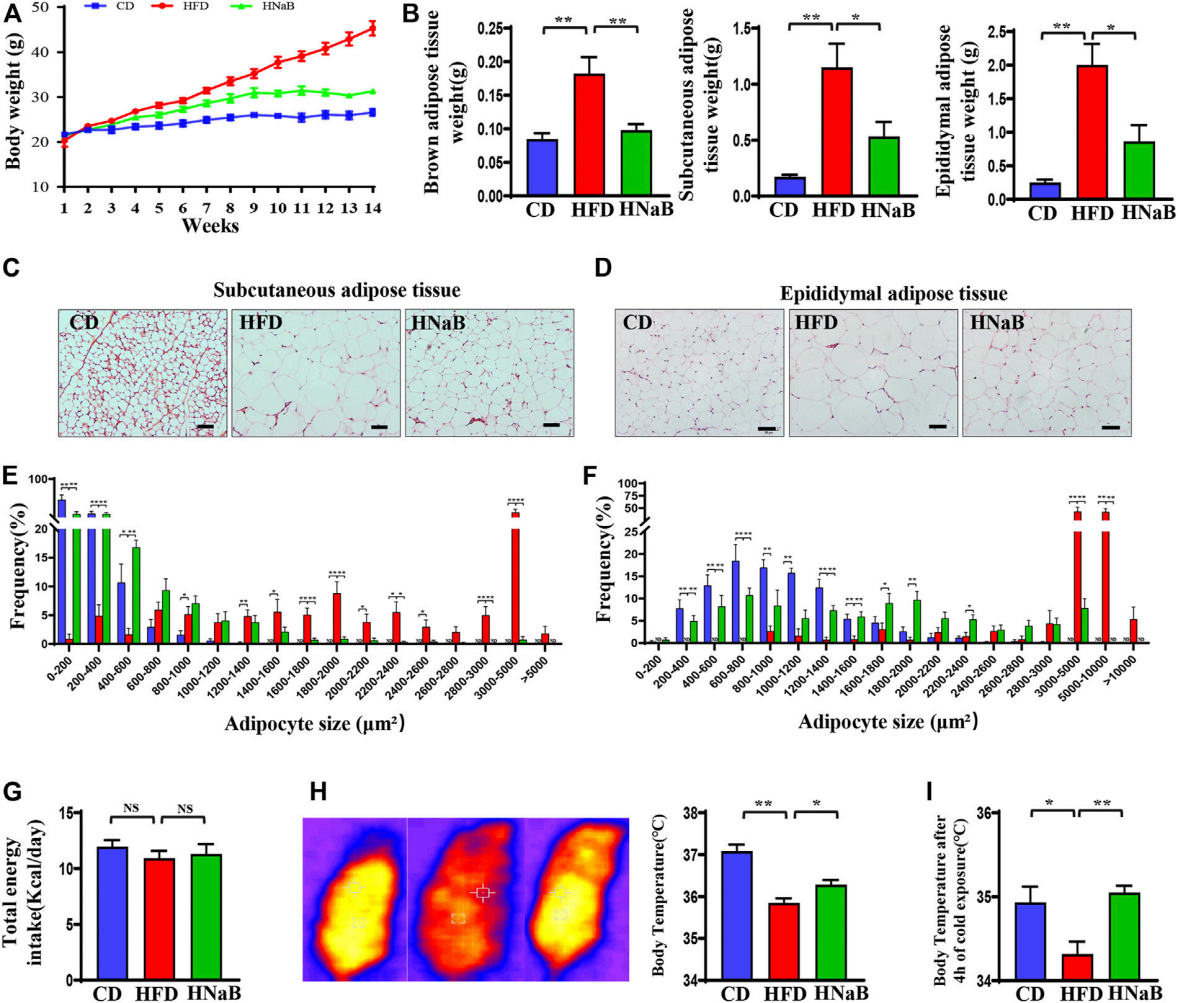
Effects of NaB on body weight under HFD. **(A)** The growth curves of mice under HFD (*n* = 5–6); **(B)** the weight of brown adipose tissue, subcutaneous adipose tissue and epididymal adipose tissue (*n* = 5–6); **(C,D)** H&E staining of subcutaneous adipose tissue and epididymal adipose tissue sections, scale bar = 50 μm; **(E,F)** Frequency distribution of adipocyte cell size in subcutaneous adipose tissue and epididymal adipose tissue (*n* = 5–6); **(G)** Daily calorie intake in mice (*n* = 5–6); **(H)** Infrared imaging of core body temperature in mice at 22°C (*n* = 5–6); **(I)** Core body temperature in mice at 4°C cold exposure (*n* = 5–6). Values are represented as the mean ± SE. One-way ANOVA with Tukey’s post hoc test, ∗*p* < 0.05, ∗∗*p* < 0.01 respectively compared with the HFD group.

To explore the effect of dietary supplementation of NaB on diet-induced obesity, the body weight of mice was measured weekly in this study. As shown in Figure 1A, the HFD mice gradually showed a difference in body weight after 3 weeks of feeding compared to the mice on chow diet. At 14 weeks, mice in the HFD group displayed an obese phenotype, dietary supplementation of NaB protected mice from diet-induced obesity (Figure 1A). Then, all mice were sacrificed and the adipose tissues were isolated. The weight of inguinal subcutaneous adipose tissue (sWAT), epididymal adipose tissue (eWAT) and brown adipose tissue in mice from the CD and NaB groups was significantly lower than that from the HFD group (Figure 1B). Obesity is characterized by an excessive accumulation of WAT, which expands by adipocyte hypertrophy and hyperplasia. Therefore, to evaluate the effect of NaB on white fat, HE staining was performed. Compared with control group, adipocytes in the HFD group exhibited round cell borders and larger cell area (Figures 1C,D). However, this phenotype was effectively ameliorated by NaB treatment, and multilocular beige adipocytes was observed in sWAT and eWAT from mice in the NaB group (Figures 1C,D). Moreover, NaB-treated mice exhibited higher proportion of small size adipocytes than that of mice on HFD (Figures 1E,F), which suggested that NaB might have potential effects on dietary obesity and fat deposition.

We next performed experiments to investigate the relevant mechanisms. As shown in Figure 1G, slight or no changes was observed in food intake among groups, meanwhile, NaB-treated mice exhibited a higher core body temperature at room temperature compared with mice on HFD (Figure 1H). Furthermore, compared with mice on chow diet, HFD-fed mice showed a rapid decrease in temperature when subjected to acute cold exposure, but this trend was diminished by NaB (Figure 1I). Together, these data suggested that the decreased adiposity in NaB-treated mice might be due to the enhanced energy expenditure.

### Effects of NaB on white adipose tissue in DIO

**FIGURE 2:**
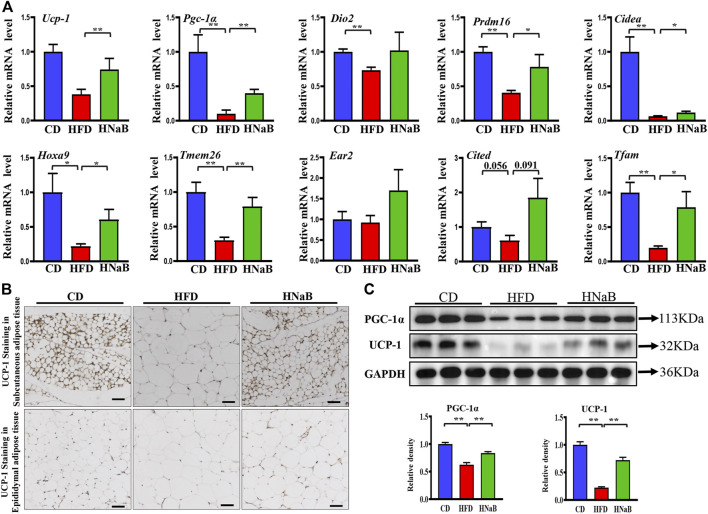
Effects of NaB on white adipose tissue in DIO. **(A)** mRNA expression of thermogenic genes and specific beige-selective genes (*n* = 5–6); **(B)** immunohistochemical staining of UCP-1 in subcutaneous adipose tissue and epididymal adipose tissue, scale bar = 50 μm; **(C)** Protein levels of PGC-1α and UCP-1 in subcutaneous adipose tissue (*n* = 3, representative of three biological replicates for each group). Relative intensity was calculated by using ImageJ software. Values are represented as the mean ± SE. One-way ANOVA with Tukey’s post hoc test, ∗*p* < 0.05, ∗∗*p* < 0.01 respectively compared with the HFD group.

Given that adipose tissue is a major organ for energy expenditure, we then performed a chain of experiments to investigate the effects of NaB on fat metabolism. qRT-PCR analysis of sWAT showed that the mRNA expression of genes involved in thermogenesis including *Ucp-1* and *Pgc-1α*, specific beige-selective genes including *Hoxa9* and *Tmem 26*, were significantly increased in sWAT from NaB-treated mice (Figure 2A). In addition, the decreased expression of *Tfam* (an important mitochondrial biogenesis marker) induced by HFD was also abolished largely by NaB treatment (Figure 2A). Immumohistochemical staining and Western blot analysis further identified that UCP-1 and PGC-1α protein levels were also upregulated by NaB in sWAT (Figures 2B,C). Despite not being considered to be the main organ for thermogenesis, eWAT is also shown to play a regulatory role in energy metabolism (Wan et al., 2014). Consistently, the protein levels of UCP-1 and PGC-1α in eWAT were also upregulated by NaB treatment (Supplementary FigureS1). These results suggested that NaB can promote the browning of WAT and energy expenditure in DIO.

### Effects of NaB on Brown adipose tissue in DIO

**FIGURE 3:**
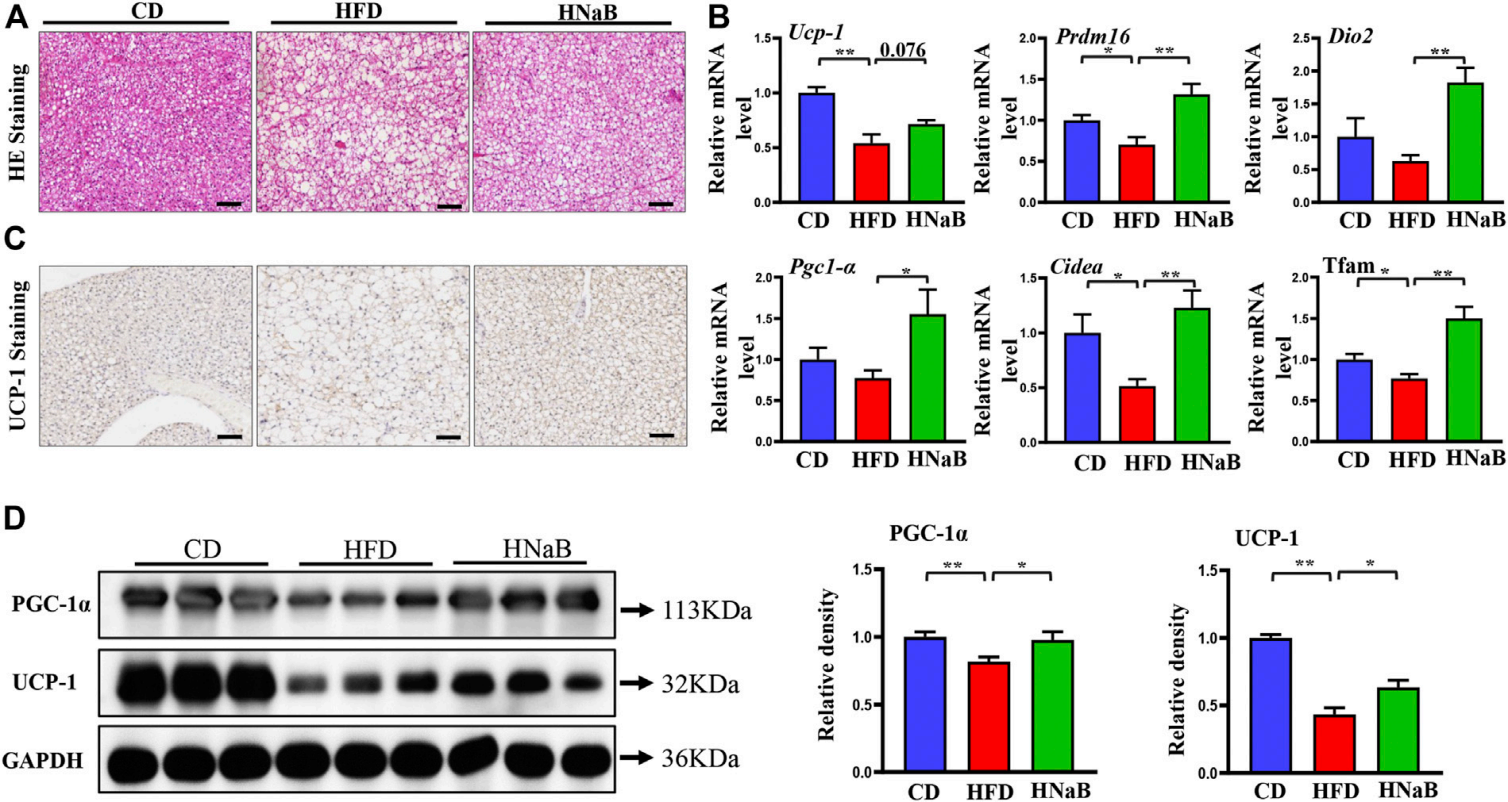
Effects of NaB on brown adipose tissue in DIO. **(A)** H&E staining, scale bar = 50 μm; **(B)** mRNA expression of thermogenic genes in brown adipose tissue (*n* = 5–6); **(C)** immunohistochemical staining of UCP-1 in interscapular brown adipose tissue, scale bar = 50 μm; **(D)** Protein levels of PGC-1α, UCP-1 in brown adipose tissue (*n* = 3, representative of three biological replicates for each group). Relative intensity was calculated by using ImageJ software. Values are represented as the mean ± SE. One-way ANOVA with Tukey’s post hoc test, ∗*p* < 0.05, ∗∗*p* < 0.01 respectively compared with the HFD group.

It is well documented that obesity is usually accompanied with disorders of adipose tissue function, lipid accumulation often results in the “whitening” of brown fat and impaired thermogenesis (Lee C. C. et al., 2019). We next performed experiments to determine whether the function of brown adipose tissue can be influenced by NaB. Indeed, HE staining analysis showed that compared with mice on CD, the area of brown adipocyte in mice from HFD group was increased obviously. Meanwhile, similar to white adipocytes, multilocular brown adipocytes were remodeled into unilocular adipocytes (Figure 3A). In order to further assess the mechanism of NaB on brown fat, we detected the mRNA expression of thermogenic genes. Compared with the CD group, thermogenic genes including *Ucp-1*, *Prdm16* and *Pgc-1α* were down-regulated significantly. However, NaB significantly restored these expression abnormalities (Figure 3B). Moreover, NaB treatment also promoted the protein expression of UCP-1 and PGC-1α of brown fat (Figures 3C,D). These data indicated that the activity of brown fat was enhanced by NaB treatment.

### Effects of NaB on sympathetic innervation of adipose tissue

**FIGURE 4:**
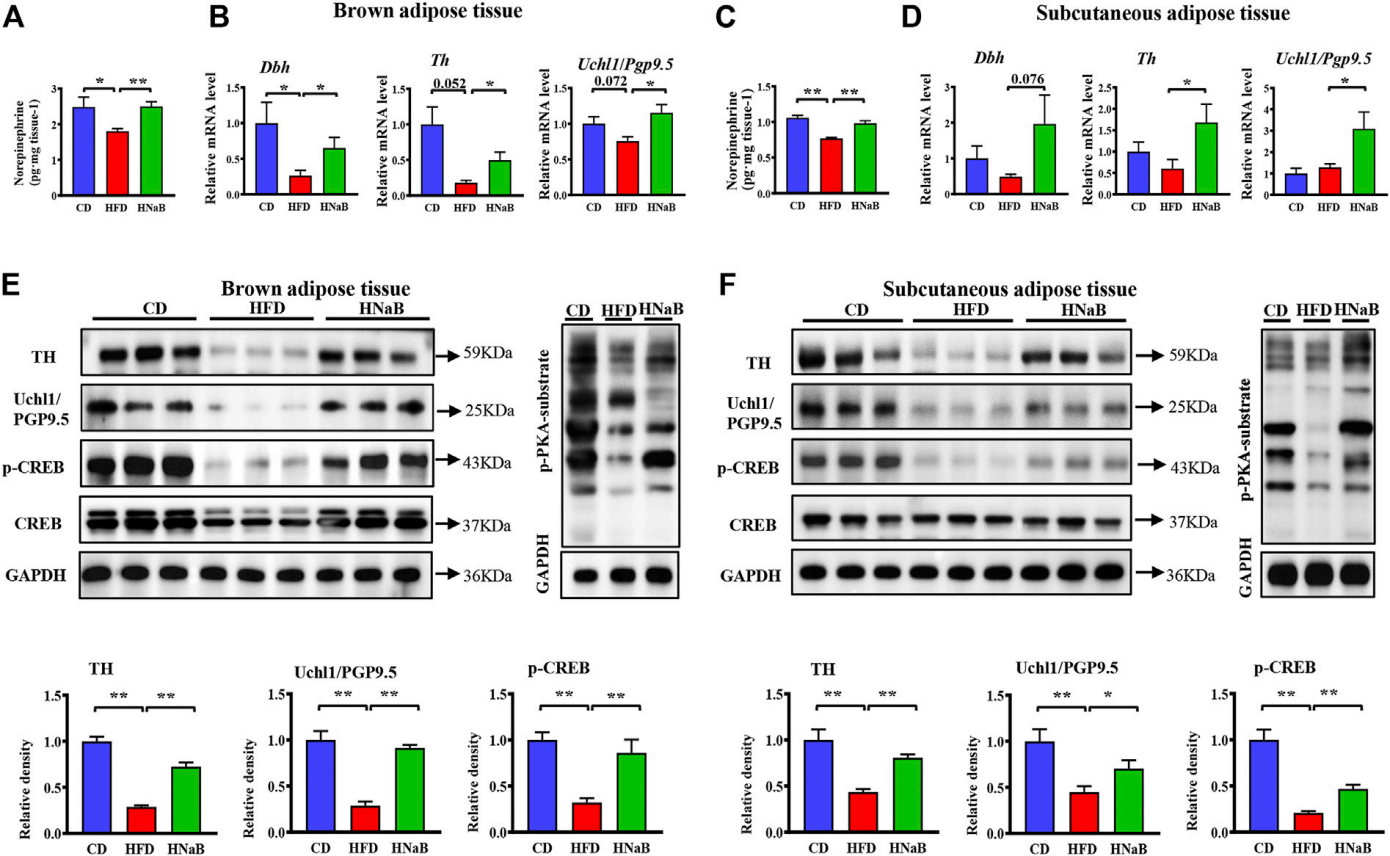
Effects of NaB on sympathetic innervation of adipose tissue. **(A–C)** Levels of norepinephrine in brown adipose tissue and subcutaneous adipose tissue (*n* = 5–6); **(B–D)** mRNA expression of *Dbh*, *Th*, and *Uchl1*/*Pgp9.5* in brown adipose tissue and subcutaneous adipose tissue (*n* = 5–6); **(E,F)** Protein levels of TH, Uchl1/PGP9.5, CREB, p-CREB, and p-PKA-substrate in brown adipose tissue and subcutaneous adipose tissue (*n* = 3, representative of three biological replicates for each group). Relative intensity was calculated by using ImageJ software. Values are represented as the mean ± SE. One-way ANOVA with Tukey’s post hoc test, ∗*p* < 0.05, ∗∗*p* < 0.01 respectively compared with the HFD group.

It has been shown that fat lipolysis and thermogenesis is mediated by sympathetic neurons that innervate adipocytes. To assess whether the effect of NaB on thermogenesis was attributable to sympathetic outputs onto the adipose tissue, we performed Elisa analysis and found the levels of key sympathetic neurotransmitters NE in both sWAT and BAT were significantly increased in NaB-treated mice (Figure 4A and Figure 4C). mRNA and protein levels of tyrosine hydroxylase (TH), a marker for sympathetic neurons, were upregulated in both sWAT and BAT from NaB-treated mice (Figure 4B and Figure 4D). Additionally, the expression of ubiquitin carboxyl-terminal esterase L1 (Uchl1/PGP9.5), another pan-neuronal marker, was also increased by NaB treatment (Figure 4B and Figure 4D). Moreover, dietary supplementation of NaB led to higher mRNA levels of dopamine β-hydroxylase (*Dbh*, an enzyme required for catecholamine synthesis) compared with HFD mice (Figure 4B and Figure 4D). Together, the results provide evidence that activity of adipose sympathetic nerves was enhanced by NaB. Effects of the SNS on fat metabolism is mainly mediated *via* cAMP-PKA signaling pathway, which induces a chain of cascade reactions likely the phosphorylation of CREB, ultimately enhances the transcriptional activity of *Pgc-1α* and *Ucp-1*. Here, we observed increased phosphorylation of PKA substrate and CREB in sWAT and BAT from NaB -treated mice (Figures 4E,F). Accordingly, the activation of BAT and browning of WAT induced by NaB might be, at least partly, *via* the increased intro-adipose sympathetic innervation.

**FIGURE 5:**
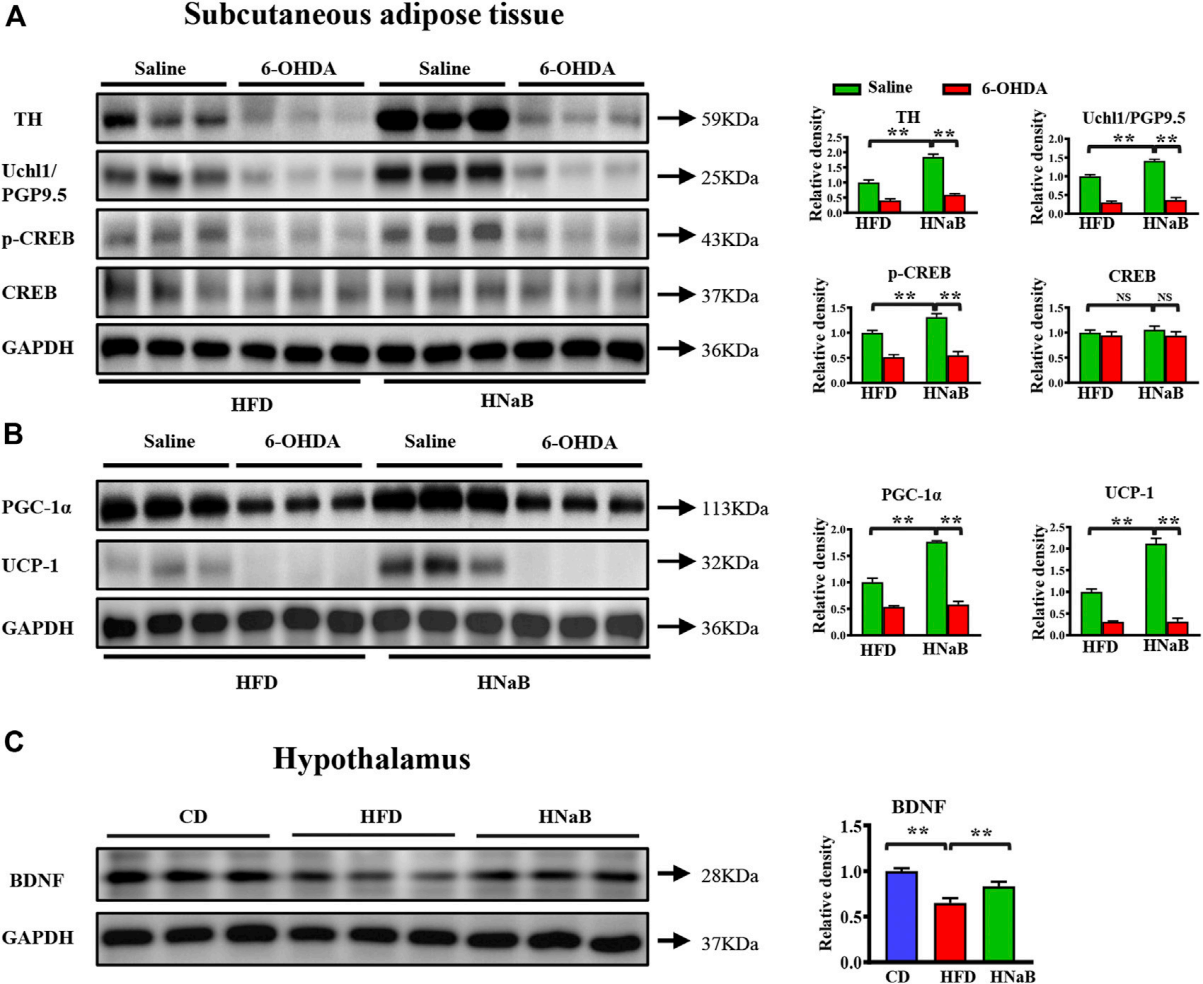
NaB enhances sympathetic nerve activity. Subcutaneous adipose tissue of mice under HFD was locally treated with saline or 6-OHDA. **(A)** Western blotting analysis of TH, Uchl1/PGP9.5, CREB, p-CREB in subcutaneous adipose tissue (*n* = 3, representative of three biological replicates for each group); **(B)** Western blotting analysis of PGC-1α, UCP-1in subcutaneous adipose tissue (*n* = 3, representative of three biological replicates for each group); **(C)** Western blotting analysis of BDNF in hypothalamus (*n* = 3, representative of three biological replicates for each group). Values are represented as the mean ± SE. One-way ANOVA with Tukey’s post hoc test, ∗*p* < 0.05, ∗∗*p* < 0.01 respectively compared with the HFD group.

To further comfirm the observed phenotype is related to enhanced sympathetic innervation, we applied the “unilateral denervation model”. Selective local sympathetic denervation is accomplished by intra-sWAT microinjections of the catecholamine-specific neurotoxin 6-hydroxy-dopamine (6-OHDA) (Rooks et al., 2005; Foster and Bartness, 2006; Giordano et al., 2006). We found that the increased protein levels of thermogenic regulators induced by NaB were abolished when blocking the β3-adrenergic signaling pathway by 6-OHDA (Figures 5A,B). In addition, it has been indicated that Brain-derived neurotrophic factor (BDNF) neurons in hypothalamus can drive thermogenesis by regulating sympathetic innervation of adipose tissue (An et al., 2015; Wang P. et al., 2020). By performing western blot analysis, NaB treatment significantly enhanced the protein levels of BDNF in hypothalamus (Figure 5C). These data indicated that the upregulated BDNF in hypothalamus might be involved in the increased intro-adipose sympathetic innervation induced by NaB.

### Effects of NaB on insulin resistance and inflammation in DIO

**FIGURE 6:**
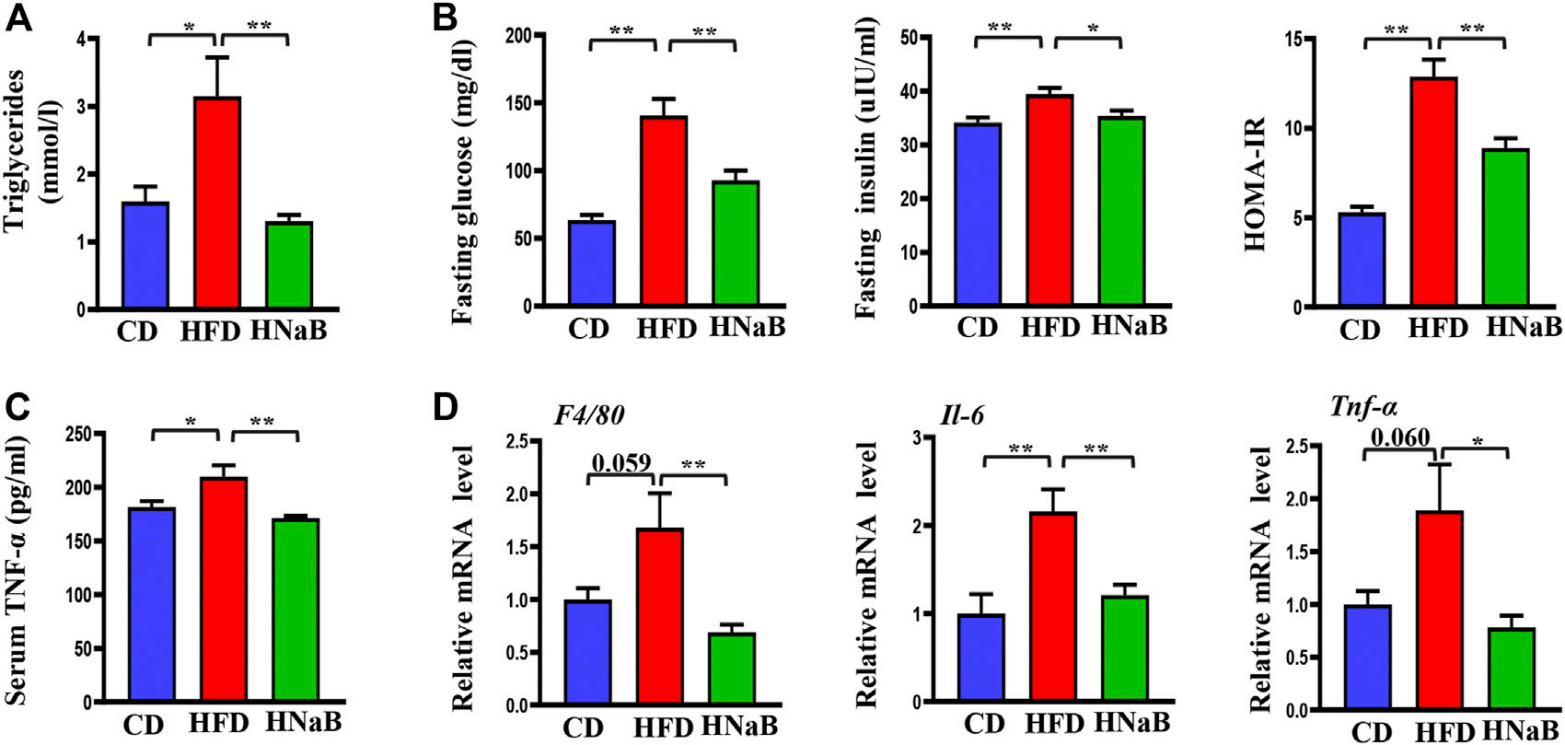
Effects of NaB on inflammation and insulin resistance in DIO. **(A)** Serum levels of triglyceride (*n* = 5–6); **(B)** Plasma glucose, insulin and the value of HOMA-IR (*n* = 5–6); **(C)** Serum TNF-α levels (*n* = 5–6); **(D)** mRNA expression of inflammatory cytokines including *F4/80*, *Il-6*, and *Tnf-α* in subcutaneous adipose tissue (*n* = 5–6). Values are represented as the mean ± SE. *n* = 5–6. One-way ANOVA with Tukey’s post hoc test, ∗*p* < 0.05, ∗∗*p* < 0.01 respectively compared with the HFD group.

Obesity is characterized by insulin resistance and low-grade chronic inflammation, we next measured the levels of plasma glucose, triglyceride and total cholesterol, and found that dietary supplementation of NaB can effectively improve the status of hyperglycemia and hypertriglyceridemia in obese mice (Figures 6A,B). To explore the effect of NaB on insulin sensitivity, we evaluated fasting insulin levels and calculated the value of homeostasis model assessment of insulin resistance (HOMA-IR) in all mice, and found that NaB treatment significantly reversed the hyperinsulinemia induced by HFD and improved insulin resistance (Figure 6B).

To further address the role of NaB on inflammation, we next performed Elisa analysis to detect the pro-inflammatory factors. The results showed that compared with the control mice, the serum TNF-α levels of mice on HFD were increased, while this effect was diminished by NaB(Figure 6C). Moreover, increased adiposity, especially in the visceral, is reported to directly cause an increase in systemic inflammation. We then performed qRT-PCR analysis on subcutaneous white fat and found that the mRNA expression levels of inflammatory cytokines such as *Tnf-α*, *F4/80*, and *IL-6* were distinctly increased in DIO. However, dietary supplementation of NaB effectively inhibited these aberrant expressions **(** Figure 6D **)**. All these data indicated that NaB can effectively attenuate inflammation and insulin resistance in DIO.

### Effects of NaB on fatty liver and gut barrier function in DIO

**FIGURE 7:**
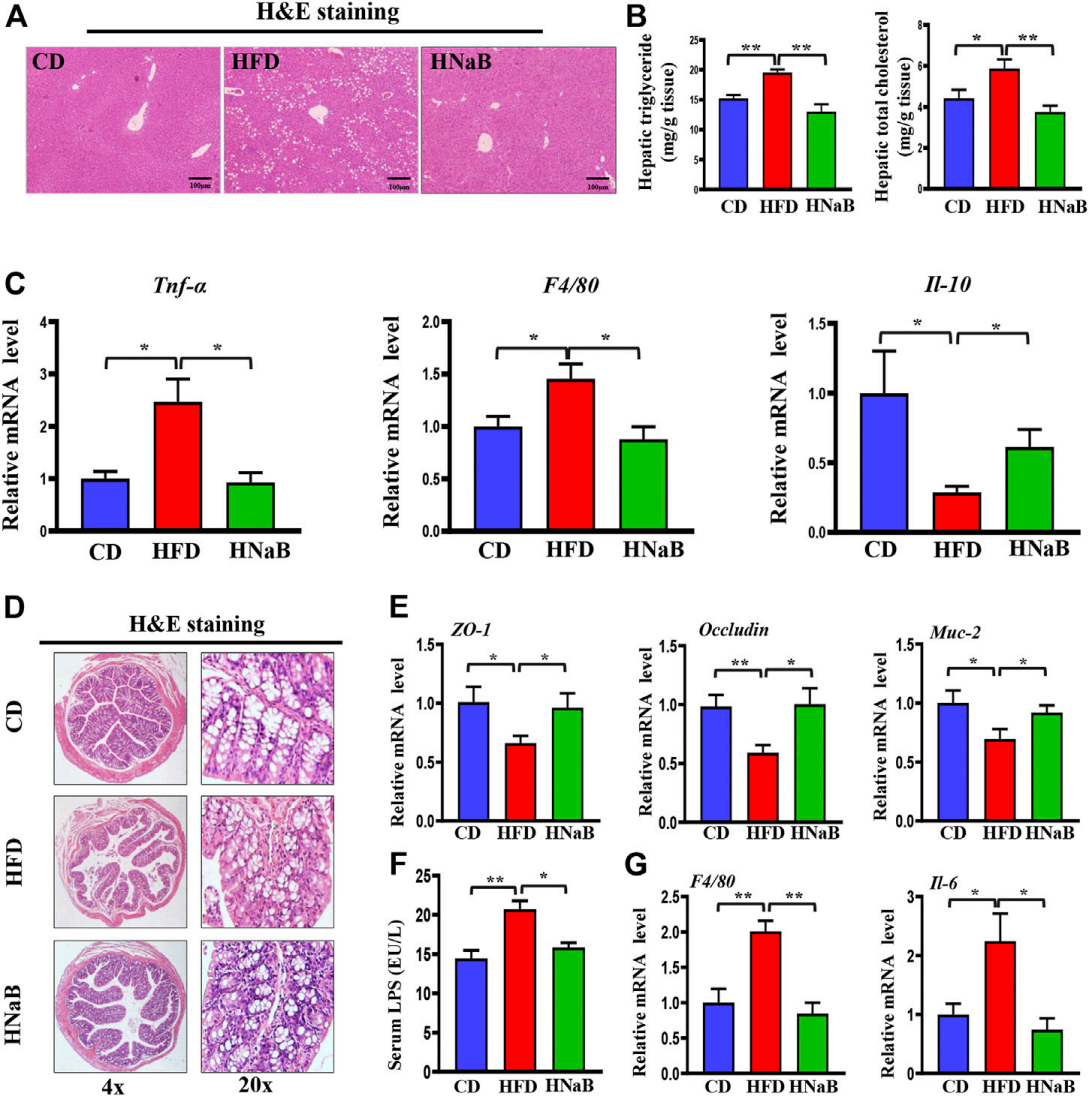
Effects of NaB on fatty liver and gut barrier function in DIO. **(A)** H&E staining of the liver, scale bar = 100 μm; **(B)** levels of triglyceride and total cholesterol in liver (*n* = 5–6). **(C)** mRNA expression of proinflammatory genes (*Tnf-α*, *F4/80*) and anti-inflammatory gene (*Il-10*) in liver; **(D)** H&E staining of colon (*n* = 5–6); **(E)** mRNA expression of *Z O -1, Occludin*, and *Muc-2* in colon; **(F)** Serum LPS levels (*n* = 5–6); **(G)** mRNA expression of inflammatory gene (*F4/80* and *Il-6*) in colon (*n* = 5–6). Values are represented as the mean ± SE. One-way ANOVA with Tukey’s post hoc test, ∗*p* < 0.05, ∗∗*p* < 0.01 respectively compared with the HFD group.

Fat metabolism disorders are shown to cause fatty liver and intestinal barrier dysfunction. In our results, the hepatocytes in the HFD group were swollen and exhibited increased lipid vacuoles, and dietary addition of NaB can effectively attenuate fat deposition in the liver (Figure 7A). Meanwhile, total liver lipids were determined and hepatic triglyceride and total cholesterol levels were found to be decreased by NaB (Figure 7B). Fat accumulation in the liver results in oxidative stress and the release of inflammatory cytokines. By performing qRT-PCR analysis of liver, add-on treatment of NaB significantly decreased the expression of pro-inflammatory gene (*Tnf-α* and *F4/80*) and increased the expression of anti-inflammatory gene (*Il-10*) compared with mice on HFD (Figure 7C).

HFD has once been shown to damage the intestinal epithelial barrier and trigger intestinal leakage. In this study, H&E staining demonstrated that mice on HFD displayed blunted colon villi and elongated crypts (Figure 7D), moreover, the expression of tight junction protein zonula occludens-1 (ZO-1), mucoprotein-2 (MUC-2) and *Occludin* in the colon from HFD mice was decreased, while NaB treatment ameliorated these phenotypes (Figure 7E). Impaired barrier function increases intestinal permeability and thereby facilitates the translocation of microbiota-derived endotoxins such as LPS into the systemic circulation (Ma et al., 2020). Here, increased serum LPS induced by HFD was abolished by NaB treatment (Figure 7F). qRT-PCR analysis further showed that NaB treatment decreased the abnormal upregulated expression of inflammatory factors (*F4/80* and *Il-6*) in colon induced by HFD (Figure 7G). All these data demonstrated that dietary supplementation of NaB can ameliorate fatty liver and gut barrier dysfunction in DIO.

## Discussion

Obesity is a high risk factor of multiple metabolic and cardiovascular diseases (Merry et al., 2020). In recent years, flora-metabolites such as SCFAs and bile acids that are reported to be beneficial for metabolic disorders (Zhang et al., 2021). As a major component of SCFAs, butyrate has been shown to effectively improve glucose and lipid metabolism in diet-induced obesity (Beisner et al., 2021). In Beisner *et al*’study, the combination of inulin and NaB can improve barrier function induced by western diet and antimicrobial peptide function in the ileum, and ultimately reduce weight gain and liver weight (Beisner et al., 2021). Meanwhile, Fang *et al* reported that NaB treatment effectively remodeled intestinal microflora structure and attenuated HFD-induced intestinal barrier, leading to decreased serum LPS levels (Fang et al., 2019). In this study, we observed that NaB can promote the activation of brown adipocytes and browning of white adipocytes, which ultimately leads to increased energy expenditure and improvement in insulin resistance, fatty liver, and intestinal dysfunction. Furthermore, these beneficial effects of NaB on thermogenesis is mediated in part through the sympathetic innervation of adipose tissue.

In previous studies, NaB have been thought to function principally in the gut. However, other study has shown that long-term dietary supplementation of NaB can facilitate this little molecule entering the blood circulation, and act directly on brain or adipose tissue (Dalile et al., 2019). Li *et al* further demonstrated that butyric acid is able to participate in the tricarboxylic acid cycle of brown and white fat (Li et al., 2019), which provides the possibility that NaB directly regulates the fat metabolism. Additionally, a recent study revealed that butyrate gavage effectively reversed the thermogenic functional impairment in antibiotic cocktail (ABX) -treated mice, which acted mainly through the activation of lysine specific demethylase in BAT and sWAT(Wang D. et al., 2020). In the current study, we found that the thermogenic effect of NaB is associated with the action of intro-adipose sympathetic nerve fibers, which uncovered a novel regulatory mechanism of butyrate in glucose and lipid metabolism. It is worth noting that besides adipose tissue, other metabolic tissues such as skeletal muscle and pancreas are also involved in the regulation of energy homeostasis (Jouvet and Estall, 2017; Balakrishnan and Thurmond, 2022), whether these tissues play a regulatory role in NaB-induced metabolic benefits remain to be determined.

Butyrate entered in systemic circulation can be distributed to the brain, and influence the expression of multiple neurotrophic factors to play regulatory roles (Petry et al., 2016; Sun et al., 2016). For instance, NaB stimulates cell proliferation and differentiation in the dentate gyrus, and enhances the expression of BDNF, prevents the impairing effects of hippocampal gastrin-releasing peptide receptor antagonism on memory consolidation and extinction (Lee H. J. et al., 2019). BDNF is also reported to be a key regulator of energy balance, and BDNF neurons in medial and posterior paraventricular hypothalamus drive thermogenesis by projecting to spinal cord and forming polysynaptic connections to brown adipose tissues (An et al., 2015). Meanwhile, a leptin-BDNF pathway was also reported to regulate sympathetic innervation of adipose tissue (Wang P. et al., 2020). In this study, the protein levels of BDNF in hypothalamus was significantly enhanced by NaB treatment. Thus, we can speculate that NaB might promote sympathetic innervation of adipose tissue, at least partly, *via* the increased expression of BDNF in hypothalamus, and further experiments are needed to explore the intricate mechanisms.

It is well documented that obesity is a chronic and complex metabolic disease that involves multiple organs and tissues, and is associated with numerous health risks including cardiovascular disease, diabetes, fatty liver and gut dysfunction (Patterson et al., 2016; Wu and Ballantyne, 2020). Insulin resistance and inflammation play a vital role in the development of above obesity-related diseases (Wu and Ballantyne, 2020). Meanwhile, it has been shown that increased fat thermogenesis can improve insulin sensitivity and metabolic disease by regulating glucose and lipid metabolism (Brandão et al., 2021). In this study, we found that administration of NaB led to an activation of brown adipocytes and browning of white adipocytes, which contributed to the improvement of insulin resistance and inflammation. This may, at least partially explain the NaB-induced benefits in liver and gut function. However, whether the increased intro-adipose sympathetic activity directly affect liver or gut remain to be determined.

In summary, our study shows that chronic NaB treatment attenuated DIO and its associated metabolic disorders by promoting fat thermogenesis *via* the increased intro-adipose sympathetic innervation, which provides a new perspective for further investigating the pharmacological action of NaB in energy metabolism.

## Data Availability

The raw data supporting the conclusions of this article will be made available by the authors, without undue reservation.

## Glossary

NaB: Sodium butyrate
HFD: high-fat diet
RT-PCR: quantitative real-time polymerase chain reaction analyses
UCP-1: uncoupling protein-1
PGC-: 1αperoxisome proliferator-activated receptor gamma coactivator-1α
Prdm16: positive regulatory domain containing 16
WAT: white adipose tissue
BAT: brown adipose tissueβ3AR, β3‐adrenergic receptor
SNS: sympathetic nervous system
NE: norepinephrine
cAMP: cyclic adenosine monophosphate
PKA: cAMP-dependent protein kinase A
CREB: cAMP-response element binding protein
SCFAs: short-chain fatty acids
CD: chow diet
TNF-α: tumor necrosis factor-α
LPS: Lipopolysaccharide
PBS: phosphate-buffered salineTfammitochondrial transcription factor A
DIO: diet-induced obesity
sWAT: inguinal subcutaneous adipose tissue
eWAT: epididymal adipose tissue
6-OHDA: 6-hydroxy-dopamine
BDNF: brain-derived neurotrophic factor
HOMA-IR: homeostasis model assessment of insulin resistance
IL-6: Interleukin- 6
ABX: antibiotic cocktail
IR: insulin resistance
TH: tyrosine hydroxylase
Uchl1/PGP9.5: ubiquitin carboxyl-terminal esterase L1
Dbh: dopamine β-hydroxylase
ZO-1: zonula occludens-1
MUC-: mucoprotein-2.
