# Supplementary material for: Sodium butyrate attenuated diet-induced obesity, insulin resistance and inflammation partly by promoting fat thermogenesis via intro-adipose sympathetic innervation
Source: Front Pharmacol. 2022 Oct 3;13:938760. doi: 10.3389/fphar.2022.938760 (PMC9574364; doi:10.3389/fphar.2022.938760)
Supplement: Supplementary file 1 [file DataSheet1.docx]

**Supplementary information**

**Supplementary table 1. List of primers sequences for qRT-PCR analysis**

| **Gene** | **Fwd (5’-3’)** | **Rev (5’-3’)** |
| --- | --- | --- |
| ***mUcp-1*** | TCTCAGCCGGCTTAATGACTG | GGCTTGCATTCTGACCTTCAC |
| ***m******Pgc-1α*** | GCACCAGAAAACAGCTCCAAG | CGTCAAACACAGCTTGACAGC |
| ***mPrdm16*** | ACACGCCAGTTCTCCAACCTGT | TGCTTGTTGAGGGAGGAGGTA |
| ***mCidea*** | TCCTATGCTGCACAGATGACG | TGCTCTTCTGTATCGCCCAGT |
| ***mCited1*** | AACCTTGGAGTGAAGGATCGC | GTAGGAGAGCCTATTGGAGATGT |
| ***mDio2*** | CATTGATGAGGCTCACCCTTC | GGTTCCGGTGCTTCTTAACCT |
| ***mHoxa9*** | CCCCGACTTCAGTCCTTGC | GATGCACGTAGGGGTGGTG |
| ***mTmem26*** | AGGGGCTTCCTTAGGGTTTTC | CCGTCTTGGATGAAGAAGCTG |
| ***mEar2*** | CCACAAAGCAGACAGGGAAAC | GCATGAGGCAAGCATTAGGAC |
| ***mTfam*** | ATTCCGAAGTGTTTTTCCAGCA | TCTGAAAGTTTTGCATCTGGGT |
| ***mTh*** | CCAAGGTTCATTGGACGGC | CTCTCCTCGAATACCACAGCC |
| ***mUchl1*** | GATGCTGAACAAAGTGTTGGC | GGAGTTTCCGATGGTCTGCTT |
| ***mDbh*** | GCTCTGTATGACTACGCCCC | AGGCTGCAGATTCCACTCAC |
| ***mTnf-α*** | GGCGGTGCCTATGTCTCA | AGGGTCTGGGCCATAGAA |
| ***mMcp-1*** | GCTGGAGAGCTACAAGAGGATC | GTCAACTTCACATTCAAAGGTGC |
| ***mIl-6*** | TTCTTGGGACTGATGCTG | CTCATTTCCACGATTTCCC |
| ***mF4/80*** | TGACTCACCTTGTGGTCCTAA | CTTCCCAGAATCCAGTCTTTCC |
| ***mZO-1*** | TGGGAACAGCACACAGTGAC | GCTGGCCCTCCTTTTAACAC |
| ***mOccludin*** | AGTACATGGCTGCTGATG | CCCACCATCCTCTTGATGTGT |
| ***mMuc-2*** | CACTGCGATGCCAACGACA | GCCACTAACTGCTTGTTCACCTGTA |
| ***m18s*** | TTGACTCAACACGGGAAACC | AGACAAATCGCTCCACCAAC |

**Supplementary table 2. Primary and secondary antibodies for Western blot analysis**

| **Antibody** | **Supplier** | **Product Code** | **Species** | **Dilution** |
| --- | --- | --- | --- | --- |
| PGC1-α | HUABIO Technology | ET1702-96 | Rabbit | 1:3000 |
| UCP-1 | Abcam | ab10983 | Rabbit | 1:3000 |
| TH | HUABIO Technology | ET1612-65 | Rabbit | 1:3000 |
| Uchl1/PGP9.5 | HUABIO Technology | ET1703-22 | Rabbit | 1:3000 |
| p-CREB | Cell Signaling Technology | 9198s | Rabbit | 1:3000 |
| CREB | HUABIO Technology | ET1601-15 | Rabbit | 1:3000 |
| p-PKA-substrate | Cell Signaling Technology | 9624S | Rabbit | 1:6000 |
| GAPDH | HUABIO Technology | BM1623 | Rabbit | 1:2000 |
| Anti-Rabbit IgG | Zen Bio Technology | 511203 | - | 1:10000 |

**Epididymal adipose tissue**

**
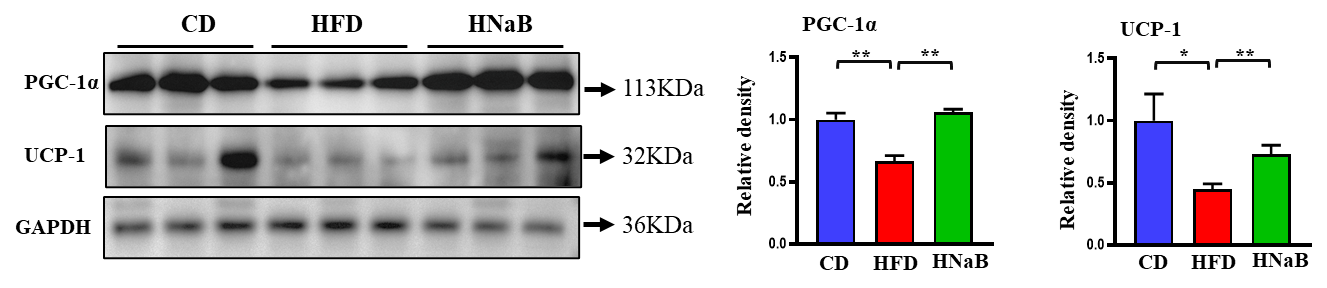
**

**Supplementary figure 1** | Protein levels of PGC-1α, UCP-1 in epididymal adipose tissue. Relative intensity was calculated by using Image J software. Values are represented as the mean ± SE (n = 3, representative of 3 biological replicates for each group). One-way ANOVA with Tukey’s post hoc test, ∗*p* <0.05, ∗∗*p* <0.01 respectively compared with the HFD group.
